# The Developmental Trajectory of a Decade of Research on Mental Health and Well-Being amongst Graduate Students: A Bibliometric Analysis

**DOI:** 10.3390/ijerph19094929

**Published:** 2022-04-19

**Authors:** Chioma Okoro, Oluwatobi Mary Owojori, Nnedinma Umeokafor

**Affiliations:** 1 Finance and Investment Management, College of Business and Economics, University of Johannesburg, P.O. Box 526, Auckland Park, Johannesburg 2006, South Africa; tobiowojori@gmail.com; 2 School of Civil Engineering and Built Environment, Liverpool John Moores University, Byrom Street, Liverpool L3 3AF, UK

**Keywords:** bibliometrics, graduate students, knowledge structure, mental health, Ph.D. students, well-being

## Abstract

The journey of graduate students through academia can be a difficult road plagued with several roadblocks due to several intersectional factors. These difficulties often impact the students’ mental health with severe consequences on their well-being and personal and academic achievements. There is a critical need for researchers to conduct studies in response to the positive mental well-being for this group of trainees, considering their peculiar role in the scholarly environment. This study aimed to explore the scientific research on the health and well-being of graduate students; typify the scientific landscape and development trajectory, cooperation networks, and fundamental research areas; and identify areas of needed research in this field. A bibliometric analysis of articles indexed in *Scopus* and published in the past decade (2012 to 2021) was undertaken. The results revealed that the research on graduate students’ mental health and well-being has increased over the years, significantly in the past two years, probably owing to the incidence of the COVID-19 pandemic and concerns around remote learning. The highest number of publications was from the United Kingdom (U.K.) and United States (U.S.), while the organizational affiliations were mainly from universities. The most prominent source type of publications was journal articles. The result also shows a weak collaboration across countries and organizations. The study identifies other areas of useful research, collaboration, intervention strategies, and policy review.

## 1. Introduction

The mental health and well-being of graduate students are of increasing concern worldwide, and though it started as an implicit recognition that students suffer poor mental health, it has expanded into an area of publicly argued concern [1]. A broad transnational survey of over 14,000 students in eight countries, including Australia, Belgium, Germany, Mexico, Northern Ireland, South Africa, Spain, and the U.S., showed that 35% of students fulfilled the criteria for one or more identified mental health conditions. It was also identified that students requiring the greatest need in terms of major distress or, in particular, poor mental health are less likely to receive support or assistance [2].

In the U.S., it has been earlier reported that there is a catastrophe of mental health amongst students across institutions of higher learning [3]. The American Psychiatric Association established a mission panel on “College Mental Health” in 2005 to provide counsel and promote investigation and intervention programs. However, this remains a problem in the U.S., while related issues concerning student mental health were also reported in Canada, Australia [4], Turkey [5], and several other nations [6].

The U.K. Royal College of Psychiatrists [7] projected, in 2011, that the degree of mental health complications among students will upsurge based on factors including the government moving many students from a broader part of the community to school at higher institutions, with increasing financial pressures on students associated with declines in public funding to sustain the students while studying. Both academic scholars and the public have shown rising apprehension regarding the well-being of graduate students, stemming from anxiety, family issues, and the extent of expectations [8]. Attention to the graduate students’ journey is critical.

In this study, graduate students are defined as individuals studying or conducting research at a higher level than a bachelor’s degree, focusing on doctorate and postdoctoral students. Doctoral students are those pursuing advanced studies beyond the master’s level to pursue an independent career, whereas post-doctoral trainees are beyond the doctoral level receiving training to pursue an independent career. These graduates are known as trainees because they receive extensive training and specialized instructions in preparation for a future in academia.

Graduate students experience a tremendous deal of stress due to high requirements, pressure, and the evaluative and competitive nature of the graduate school, which may contribute to increased stress and vulnerability [9]. In addition to their studies, graduate students often juggle other responsibilities, including supervision, teaching, or research assistance. Thus, academic and coursework difficulties, financial pressures, anxiety, and a lack of work–life balance are all stressors caused by the combined effort of these positions, leading to burnout, fatigue, depression, and physical health difficulties [10].

Studies support that graduate students face significant stress, and attention to their needs and challenges is paramount [11,12,13,14,15]. According to a survey, graduate students reported that their mental health had worsened during their education [16], while another reported that one out of every three students sought counseling for anxiety or depression over their journey through graduate school [17]. Supporting these views, Jones-White et al. [18] revealed factors contributing to graduate students’ anxiety and depressive disorders as a lack of a sense of belonging and academic, financial, and relationship stressors. One out of every three graduate students is at risk of having a mental health problem, such as depression [15]. Graduate students have a self-reported incidence of depression and anxiety six times higher than the general population and their peers in the same age bracket [14]. Therefore, they require more significant support to handle mental health concerns [19,20].

Numerous factors detrimental to students’ well-being are debatably exclusive to the postgraduate journey. Doctoral degree students struggle with emotions of social seclusion, absence of enthusiasm, difficulties with their advisors or supervisors, and loss of engagement with the educational community [12,21]. About 56% of doctoral students consider dropping out during the process due to experiences of anxiety, stress, exhaustion, and lack of interest [12]. Lately, consideration of such factors in studies has been reduced, as research has concentrated on how those elements influence their completion rate [22]. Considering the high degree of well-being hypothetically needed to accomplish a doctoral degree, it is no wonder that low well-being significantly impacts the students’ research progress, career advancement, academic efficiency, and private lives [22]. These problems have organizational and financial repercussions for higher educational institutions [12,23]. While existing studies have assessed some phases of mental health, most frequently, the aspects of psychological distress [12,15,16] and other aspects such as psychological well-being have not been well-researched.

Conventionally, mental well-being and mental distress were regarded to represent opposites of a particular dimension, with increasing mental well-being implying lower mental suffering and vice versa. Increased mental well-being has also been reported to reduce mental distress over a period, while declines in mental well-being reflect increased mental distress [24]. A variety of additional models for how mental well-being and mental distress interact to influence an individual’s mental health suggested that both mental well-being and mental distress have a role in graduate students’ overall mental health.

Therefore, there is a need to undertake continuous research on this topic to ensure that the related concerns are clearly and consistently mapped and intellectualized using appropriate and reliable tools for measurement. Seeing the high level of depression and anxiety among the doctoral student population, it is important to expand on research focused on this critical group given their job prospects and contribution to the broader society [25]. Doctoral students and postdoctoral trainees have the prospect of research, development, and education at institutions and beyond, but they are in danger of losing their jobs. The increasingly bleak job environment for scientific researchers adds to the dissatisfaction produced by long hours and low compensation. Though a bit of stress can be tolerated, anxiety and depression can be devastating. In 2021, for example, a study of doctoral students at seven United States (U.S.) universities found that 15.8% of them had considered suicide within two weeks before taking the survey. According to the survey, just one-third of those who fulfilled the diagnosis received therapy, and students with psychological problems were also more alienated, had fewer colleagues to resort to for support, and were much more inclined to consider quitting school [26]. Therefore, systematically monitoring and championing research on their mental health is paramount, ultimately contributing to increased completion rates [16,27,28]. Not recognizing and responding to this problem could result in a considerable loss of resources and human potential.

Furthermore, the COVID-19 pandemic has likely made these difficulties more severe, and graduate students may find it even more challenging to seek support [29]. Some students may be hesitant to acknowledge it, let alone manage it, because of enormous competition, exacerbating the situation. According to research by the World Health Organization (WHO) [30], the number of persons with mental health disorders is expected to rise, and graduate students are among the most vulnerable groups. Therefore, research on this topic is vital.

Although studies on the mental well-being of graduate students have been undertaken, no study has comprehensively highlighted the knowledge areas on this topic using a bibliometric review. For instance, Levecque [15] and Almasri [26] conducted their study on the prevalence of mental problems among Ph.D. students using a web-based questionnaire. Hazell et al. [31] and Jackman et al. [32] used systematic reviews, while Satinsky et al. [27] conducted systematic and meta-analysis. This study represents the only bibliometric study on the subject at the time of analysis. It is essential to track and document the trends and extent to which the subject has been addressed, i.e., hotspots and gaps, making it possible to predict emerging trends and suggest future research. Therefore, the study’s objectives were to:

1. Map the trajectory of research on graduate students’ mental health and well-being on an international scale;
2. Analyze the knowledge structure in terms of publications’ co-authorship and organizational and journal sources; and
3. Establish the existing research thematic areas, hotspots, and gaps (areas that have received inadequate attention).

This bibliometric review utilized systematic methods to identify 63 *Scopus*-indexed publications within the last decade (from 2012–2021) on graduate students’ mental health and well-being. The data extracted from these documents were analyzed quantitatively. The findings are envisaged to inform innovative research that can institute intervention measures theoretically and practically. The following section presents an overview of the conceptual framework.

## 2. Conceptual Framework

The concept of the bibliometric review was proposed in 1969 [33]. The concept argued that quantitatively analyzing patterns and boundaries offers the potential to gain broader insights into a particular field’s research work. Bibliometrics use principal tools to measure research output while providing a framework of research conducted by other actors concerning collaboration, distribution of scientific productivity, and authorship patterns [34]. It involves applying statistical techniques to define qualitative and quantitative variations in a presumed scientific subject of research [35]. This method avails relevant statistics for researchers looking to measure scientific works in an area by granting a swift understanding of the current data and identifying the new developments available in the research sphere. It further explains the association between each unit of analysis through graphics and imaging to better comprehend the research hotspots, growth, and recent research in the field to realize educated findings.

Even though some studies have reported on the literature in different subject fields, there have been no bibliometric studies explicitly conducted on graduate students’ mental health and well-being. Quantitative surveys have been undertaken on Economics graduate students’ mental health [16], the mental health crisis in graduate education [14], the link between mental health and doctoral students [15], experiences of belonging in relation to mental health and well-being [36], and the perceptions on and assessment of mental health [37,38]. Another study used critical self-reflections on postgraduate researchers’ mental health and well-being [39]. Other studies used the bibliometrics method to analyze the publications on the related subject matter, mapping university students’ mental health and well-being, including undergraduate and postgraduate [40] and international students [41]. These conceptual studies established that the frequency of mental health complications is more in doctoral students than in other higher education student populations, as supported in another study [42]. It was thought-provoking to note that these studies identified the mental health of graduate students as a continuing gap in the literature.

Diverging from the above, the current study did not concentrate on a specific set of students (e.g., international). Instead, it adopts an extensive perspective on all typologies of graduate students while acknowledging that their experiences may vary across different geographical, socio-cultural, and intersectional contexts. This bibliometric review is structured to extend literature and cover the gaps in previous studies given that the currently limited studies adopt systematized review approaches for graduate students’ mental health and well-being research. Hence, this study acts as a proposal that charts the literature on graduate students’ mental health in a systematized manner, which will expectantly decrease some of the complications deterring researchers from investigating this topic and, most importantly, open up innovative ideas and methods of mitigating the dilemma of this group of trainees (students) with the heightening emergency.

In this study, the developmental trajectory, co-occurrence analysis, and performance analysis were used exclusively to study the knowledge structure of scientific publications on the subject. The co-occurrence analysis was valuable to uncover the knowledge structure and progress of research fields. Co-word analysis was applied based on the co-word matrix, consisting of the cluster analysis and social network analysis. Co-word analysis was employed to explore conceptual work and knowledge trends across diverse domains [43]. The next part of this study is structured as follows: Section 3 describes the methodology, Section 4 presents the findings and discussion, and Section 5 provides the concluding report.

## 3. Methods

Bibliometric analysis applies statistical tools to assess research production for individuals, institutions, and countries [44]. Bibliometric methods are primarily quantitative, but they can also generate qualitative statements about scientific activities [45]. Bibliometric analysis is a helpful and effective method for obtaining information about the present status of research in specific fields, and it helps researchers quickly and easily find and pursue new lines of research [46]. As bibliometrics’ main strength, such analyses also tend to provide information on the research impact of institutions/organizations, researchers, and sectors, such as geographical sectors, and their scientific influence.

### 3.1. Search Strategies

The search for materials was undertaken in the *Scopus* database. Although other indexing databases are available, such as *Web of Science*, *Google Scholar*, and *PubMed*, for bibliometrics, this study utilized only the *Scopus* database because it is one of the largest reputable databases for retrieving related documents. The search identified the wide-ranging literature making mention of mental health and well-being in graduate students through the following query on the *Scopus* database:

“mental health and well-being” OR “psychological well-being” AND “PhD students” OR “Graduate students” OR “post-doctoral students” AND “(LIMIT-TO (PUBYEAR, 2021) OR LIMIT-TO (PUBYEAR, 2020) OR LIMIT-TO (PUBYEAR, 2019) OR LIMIT-TO (PUBYEAR, 2018) OR LIMIT-TO (PUBYEAR, 2017) OR LIMIT-TO (PUBYEAR, 2016) OR LIMIT-TO (PUBYEAR, 2015) OR LIMIT-TO (PUBYEAR, 2014) OR LIMIT-TO (PUBYEAR, 2013) OR LIMIT-TO (PUBYEAR, 2012)) AND (LIMIT-TO (OA, “all”)) AND (LIMIT-TO (LANGUAGE, “English”))”

The publication types included in the search were articles, reviews, and notes. The search was conducted on 11 November 2021. To circumvent probable bias owing to the constant update of the database, the collection and export of selected publications were performed in a day. The search produced a sum of 1722 publications in the *Scopus* database. This was further subject to the appropriate inclusion and exclusion parameters.

### 3.2. Inclusion and Exclusion Criteria

From the pool of 1722 publications identified from the *Scopus* repository, a systemic review method was employed, which is a technique directed by a set of inclusion and exclusion criteria to screen studies that are unrelated to the research questions and objectives. Firstly, the publication year was limited to the past ten years (2012–2021). Secondly, all publications that are not open-access were excluded. Thirdly, only publications that were published in the English language were selected for the analysis. Furthermore, the selection went through sifting the abstracts and titles to identify relevant studies. Sixty-three studies were deemed relevant for analysis after the screening and thus exported to the VOSviewer software for analysis.

### 3.3. Method of Analysis and Software

**Figure 1:**
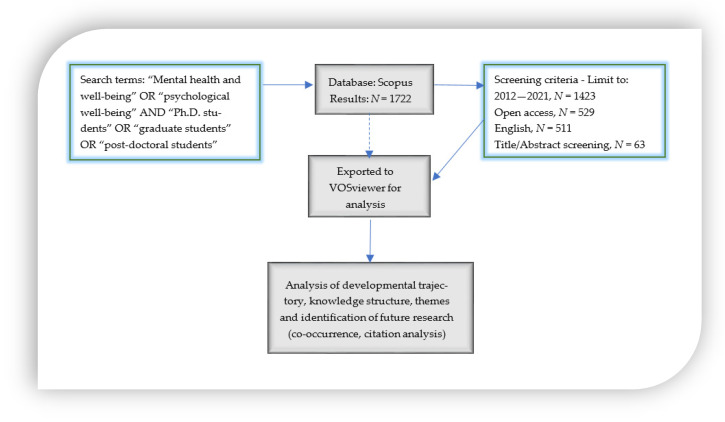
The Research Process.

VOSviewer is an established and versatile software program created by Van Eck and Waltman [47] to generate, visualize, and analyze bibliographic maps [48]. An advantage of this software is that it concentrates on imaging and graphical illustrations of maps—large ones; it creates maps based on network data and offers distinct attributes such as plotting and normalization [49]. It is also easier to interpret and infer conclusions without complications than with other visualization software such as Gephi and Bib-Excel [49]. Figure 1 presents the research process employed in this study.

## 4. Findings and Discussion

### 4.1. The Development Trajectory of Studies on Graduate Students’ Mental Health and Well-Being

**Figure 2:**
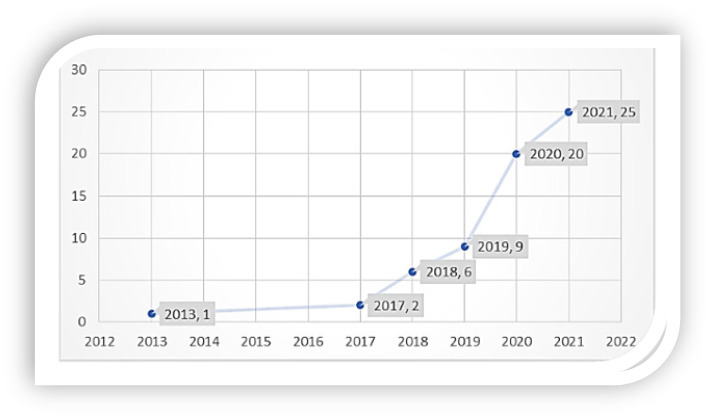
Publication Trajectory (year-wise).

The numerical growth of research publications on a subject represents a vital indicator of the evolutional trend in that research domain. It mirrors the image of development and the advancement of knowledge on the subject. By analyzing of the number of published literature over the years, the state of research and the trend of imminent development in a particular field can be clearly interpreted and understood. In 2012–2021, research on graduate students’ mental health and well-being made up 63 publications on a global scale as indexed in *Scopus*. The number of published documents on the subject gradually increases over time, as shown in Figure 2. However, publications were relatively small in this area, numbering 18 from 2013 to 2019, suggesting that there has been limited attention to the predicament of this group of people through scholarly research. The growth trend witnessed publications significantly increase from 2020–2021. Specifically, the last two years have observed a remarkable growth of research in mental health in tertiary education, particularly among graduate students. These publications have heralded new opportunities and awareness in the educational sector leading to a scholarly community of research and publishing directed to reaching a wider audience. The publications also reflect that new researchers are increasingly examining the field instead of a set of researchers dominating a particular field. These findings suggest that the patterns may have been influenced by the incidence of the COVID-19 pandemic, which birthed plenteous research on mental health and the impact of the associated restrictions on research, teaching, and learning in higher education in general. The relevance of attention to students’ mental health was elevated given the sudden shift to remote or online learning, which possibly spiked research on graduate students’ well-being.

### 4.2. Geographical Distribution

Drawing on the retrieved data, researchers from different countries have contributed to the studies on mental health and well-being among graduate students during the last ten years. The location variation and connectedness reflect the country’s contribution and demonstrate where the most significant research interest exists. In bibliometrics, geographic perspectives are helpful in analyzing the spatial and country-wise distribution of scientific publications on a specific topic and the underlying variables in each situation.

**Figure 3:**
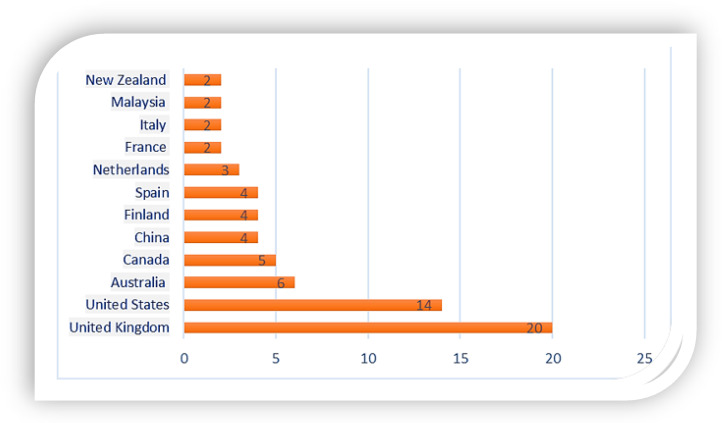
Geographical Distribution of Publications.

The generated list of the top publishing countries is shown in Figure 3. Publication outputs from the U.K. recorded the most significant number of publications (22, 7%) followed by the U.S. (15.9%), Australia (6.8%), Canada (5.7%), and Spain (4.7%). The following can be seen from the chart: Firstly, the publication strength of developed nations is more robust than that of developing and underdeveloped nations. Secondly, the publication record across countries is relatively low even though it has seen an increase in the past two years. This suggests the need for countries to invest increasingly in the mental health and well-being of their citizens, primarily through scientific research. A survey carried out by WHO’s 194 member states reported that only 52% of member countries worldwide met the given target concerning the promotion of mental health and preventive programs for citizens, which is well below the 80% target set by the WHO [50].

### 4.3. Distribution Source-Wise

**Figure 4:**
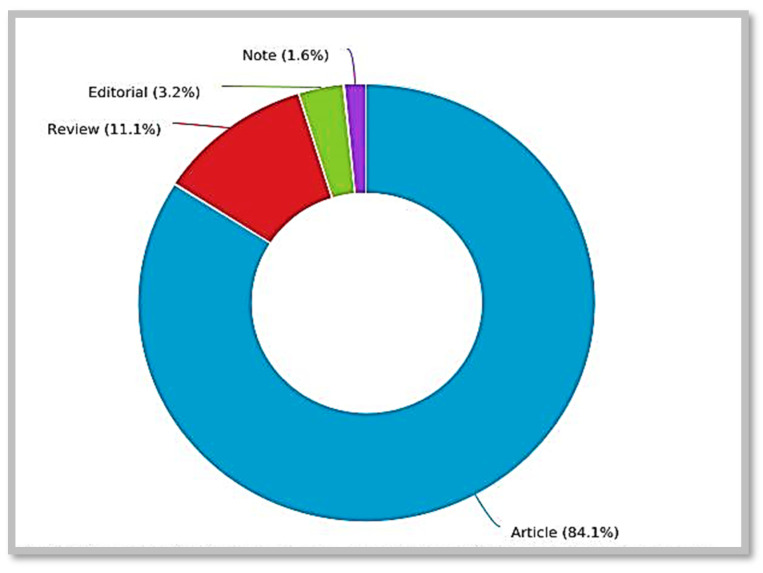
Types of Documents Retrieved.

The 63 relevant documents were retrieved from three sources, including journal articles, reviews, and notes. Figure 4 projects information concerning the different types of documents source included in the research. The research article records the highest source of publications by the researchers with 53 (84.1%) publications, followed by reviews (7, 11.1%), editorials (2, 3.2%), and notes (1, 1.6%). The result of this distribution indicates that most of the research carried out on this subject are articles via empirical or primary studies through the use of qualitative, quantitative, experimental, or mixed methods. While this is a notable source for disseminating original research, review papers also offer a firm platform for future research in that they supply understanding and extend vital insights into new areas. The notes represent the most minor publication source, which could be due to the perceived loss of information critical to the interpretation associated with notes, and many authors may not want to reduce much of the information from their findings. Notwithstanding, it has several benefits, including a brief presentation of findings, rapid gain of relevant information, and reduced publication fees [51].

### 4.4. Country-Wise Collaboration

**Figure 5:**
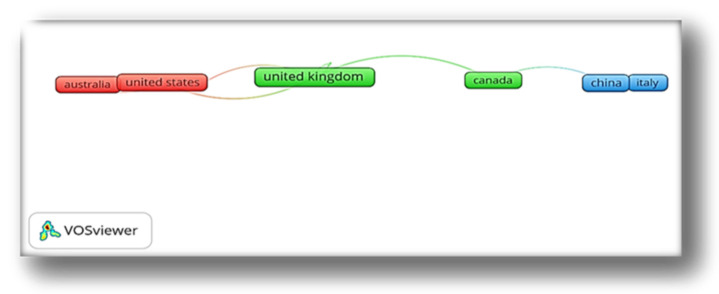
Country-level Collaboration Map of Countries.

Figure 5 shows three clusters of countries that exhibit collaboration with other countries. The red cluster indicates collaboration between the U.S., New Zealand, Australia, and France. The green cluster has the highest collaboration, showing that the U.K. closely collaborates with the USA, Canada, France, and Spain, with a total link strength of 8. The collaboration in the blue cluster is China, Italy, and the Netherlands, with a link strength of 3, 2, and 2, respectively. The collaboration strength amongst the countries is generally weak and low, suggesting that most of the countries have primarily concentrated on independent and autonomous research regarding the mental health of graduate students and show limited cooperation with other researchers. Collaboration in mental healthcare has proven to produce positive outcomes for the affected [52]. This presents a strong case for the need for collaboration.

Given the international nature of the difficulties in the development of mental health interventions, there is a need for increased global commitment, which can best be achieved through collaboration across countries. Bearing in mind that the mental health subject is sensitive to cultural, social, political individual contexts, and morals [53], collaboration increases sensitivity to these differences and builds on the knowledge of diverse communities. The experiences across countries can assist in speeding up innovative techniques in providing mental health intervention in other nations. Further, collaboration eliminates the possibility of “reinventing the wheel” by applying the knowledge forward from the experience of other nations. Lastly, funding from organizations can kick start innovative programs and initiate collaborative programs on mental health across different countries.

The differences in cultures across different countries also have a range of implications for mental health practice, ranging from how people view health and illness to treatment-seeking patterns, the nature of the therapeutic relationship, and issues of racism and discrimination. Mental health is a complex issue that requires collaboration among all stakeholders, including grassroots and community organizations, civil society groups, local and national governments, international organizations, private sector companies, religious groups, and academic institutions. Much of the theory and practice of mental health, including psychiatry and mainstream psychology, have emerged from western cultural traditions and understandings of the human condition. The concepts of the epistemology of body and mind, positivism, and naturalism have all played a role in forming today’s current mental healthcare [54,55]. Since such understandings of mental well-being have supplied powerful conceptual frameworks and tools for the relief of mental distress in many contexts, they have also proven to be problematic when adapted to non-Western cultures without considering the intricacies with which having to work across cultures tends to bring [56]. According to Tribe [57], Western cultural models to health are founded on a concept that emphasizes individual intra-personal experience or dysfunction, whereas other cultures may emphasize societal or family dynamics. There are indeed many aspects at the intersection of psychological health and culture that mental health experts must take into account if they want to actively involve the people they work with, from matters of over-representation of particular cultural groups in treatment centers to studies that exclude some cultural groups while including others [57,58].

Regarding how individuals seek care, cultures differ as well. According to research conducted in developed countries, such as Australia, Canada, and the U.S., various cultures request support considerably later than the majority population, and many of them appear in critical cases of mental distress [58]. A major reason for this can be attributed to perceived shame, as explored in some studies. Some cultures attribute the illness to the presence of spirits, the dark eye, black power, or the violation of taboos, putting the problem under the ambit of herbalists. Healing shrines in India and other holy pilgrimage regions around the world are typical examples [59]. Because of social shame, mental illness has become a hidden concern across much of Africa, equating to a silent plague. Many homes with mentally ill members hide them for fear of prejudice and ostracism from their community [60].

It is worth noting that people of different cultures may not draw a similar distinction between bodily and mental difficulties that people in Western therapeutic systems do. Feldmann et al. [61] discovered that study respondents in the Netherlands see no difference between mental and physical health. This is in stark contrast to Western psychiatry, which typically takes a reductionist approach whereby the body and mind are entirely differentiated [62]. As a result, a cross-cultural strategy that considers the needs of individual groups is vital.

### 4.5. Distribution by Discipline Area

**Figure 6:**
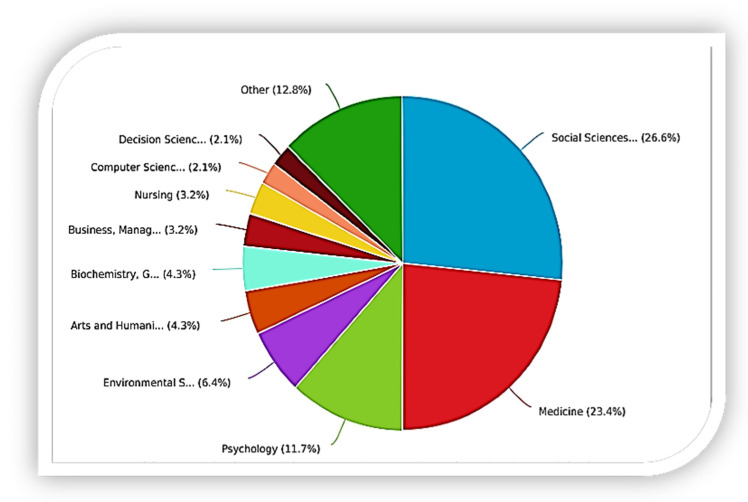
Distribution by Discipline Area.

The studies on mental health and well-being of graduate students are not confined to a specific discipline. Diverse disciplines have engaged in contributing to different aspects of the research. Figure 6 displays the subject area as defined by the retrieved studies. It was observed that social science received more attention, with a total of 17 (26.6%) publications, followed by medicine (15, 23.4%), psychology (8, 11.7%), and environmental sciences (5, 6.4%). It is unexpected that industries such as engineering and construction are not highlighted in the figure. They may be categorized in “others”, but this suggests that there is less attention in the areas when compared to others that are represented in the figure. The construction industry is known for a poor mental health and well-being record. A possible explanation for the lack of representation could be the research criteria where only studies indexed in *Scopus* under various open-access models were included in the study.

Research evidence supports multi-disciplinary team-working as a powerful and effective means of providing inclusive mental health interventions to persons with mental health challenges. With increasing specialty and growing knowledge about health and mental health conditions, having multi-disciplinary groups that replicate different areas of specialization is ever more necessary. It is envisaged that more diverse disciplines, for example, languages, arts, data science, and machine learning, will increasingly be incorporated in this area of research in the next years.

### 4.6. Organization Affiliation

**Figure 7:**
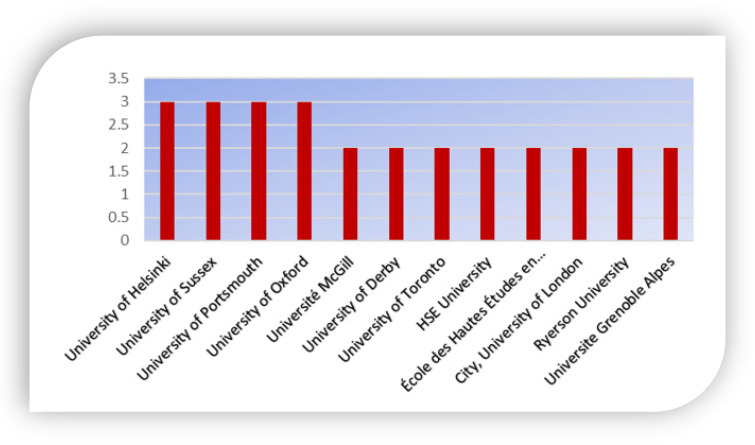
Organizational Affiliations of the Authors.

Statistics relating to organizations with greater productivity on the mental health of graduate students is presented in Figure 7. Based on the retrieved information, the top 12 organizations internationally are as shown below. The research growth of universities or institutions is an essential indicator of a nation’s capability in scientific research, innovation, and development. All organizations in the chart are universities. This is indicative of the highly enthusiastic atmosphere of research in those universities. Among the foremost twelve institutions, the majority are from Europe (the University of Helsinki, University of Sussex, University of Portsmouth, University of Oxford, and University of Derby), three of the institutions are located in Canada, and two in France. This indicates that the published literature is highly skewed toward the European countries and the university sector.

### 4.7. Main Subject Word Analysis

The keywords are regarded as a high-level synopsis of the research publications. The highly frequented keywords used by authors were used to epitomize the fundamental areas of research and the hot spots in a research domain.

**Figure 8:**
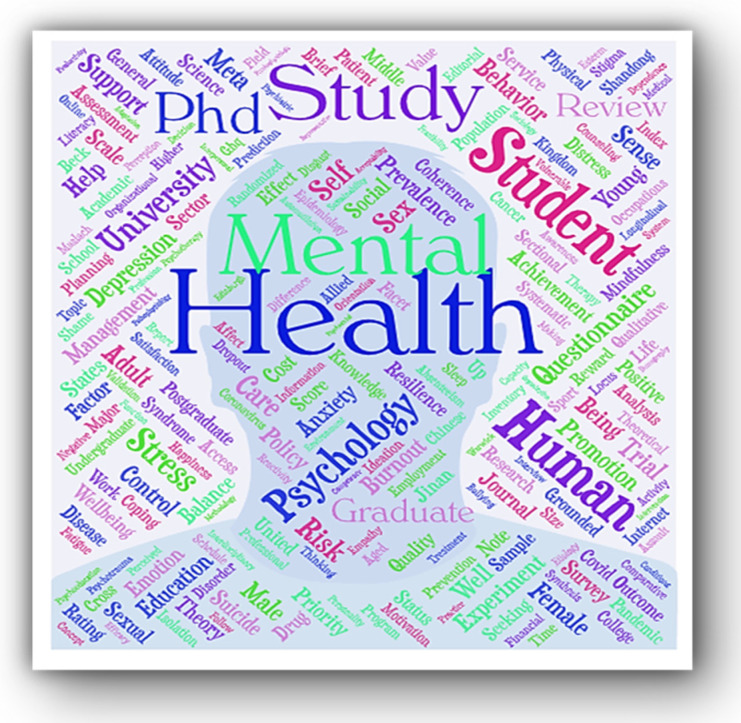
Authors’ Most Frequent Keywords.

In this study, firstly, keywords of the bibliographic dataset (*n* = 429) were explored. The five most frequently occurring terms (excludes common stop-words) included “mental health” “psychology”, “Ph.D.” “student”, and “human”. The most-occurring keywords are shown in a word cloud (Figure 8). The highest keyword co-occurrence are “mental and health” (*n* = 562) followed by “psychological and well-being” (*n* = 412), “health and promotion” (*n* = 390), “Ph.D. and student” (*n* = 352), “doctoral and student” (*n* = 323), and “social and support”. To obtain more information on the keywords, co-occurrence was visualized.

### 4.8. Analysis of Keywords

**Figure 9:**
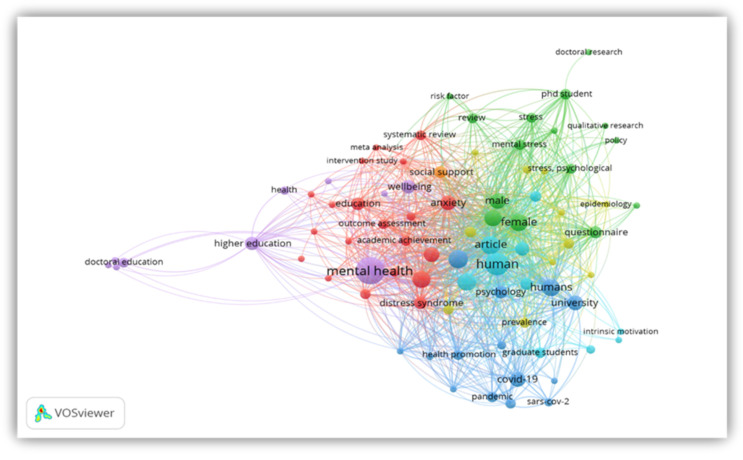
Network Visualization Map of Co-occurring Terms.

The knowledge trend was explored through the analysis of the results of the co-occurrence analysis. The VOSviewer generated a co-word map using the keyword co-occurrences (Figure 9). The analysis of keyword clusters is a quantitative measure to establish the close connection among items based on their features and certain similarity or dissimilarity pointers in the literature [49]. The keywords in each cluster were synthesized to reveal topics in the literature. The map reveals five themes representing mental health research among graduate students.

Cluster 1: The first (purple-colored) category of cluster analysis is concerned with the general perspective of mental health in higher education, encompassing all graduate students. The focus of this cluster is on comprehending the mental health of graduate researchers through the conceptualization of doctoral researchers’ mental health and the characterization of the Ph.D. experience. The studies in this cluster identified several risks and protective factors at the individual, interpersonal, and systemic levels as important in determining doctoral researchers’ mental health. The factors representing the most substantial evidence base (as supported by many of the studies via mixed-method research) indicate that being female and secluded increases the possibility of mental health issues, and envisioning the Ph.D. as a process, sense of social support, positive supervisor connection, and personal care are essential [31,63].

Cluster 2: The second (red-colored) group deals with “intervention studies, a meta-analysis on the distress syndrome among graduate students concerning their academic achievement”. The cluster identifies a need for intervention at different levels, such as instruction, prevention, care, and follow-up to lessen mental health complications amongst Ph.D. students. It also highlights the supposed role of the university in ensuring a sense of belonging to the community as a possible factor to cut these problems. The cluster provided the basis for more understanding of the concerns associated with the prompt realization of the doctorate. The self-determination theory was well-utilized as the framework for the studies. More specifically, the basic needs theory, one of the sub-theories of self-determination [64], emphasizes the need for people to meet the three basic requirements of competence, relatedness, and autonomy [64,65]. These were discussed as a concept to foster feelings of association and a sense of belonging among graduate students.

Cluster 3: The third category (green-colored) represents a summary of the risk factors and policies that contribute to the mental distress of graduate students. Graduate students experience a variety of stressors at different levels of study. These include pressure for graduation, job prospects, relationships, and others [22,66]. In addition, authors in this category identified that the dependencies between the core stress factors amongst these trainee groups in the literature are yet well-comprehended. Scholars are starting to view how parts of graduate education, such as economic uncertainty, role dispute, too much workload, unclear career visions, insufficient supervision, and absence of clarity in the institutional policies, can intersect in the perspective of mental health. The studies contained in this cluster force a re-thinking of the link between the generally existing stress and applicable guidelines and regulations and suggest counselling and review of policies. The studies here further emphasized applying social-ecological theory to demonstrate how several ecological settings have an accumulative and joint impact on well-being [67,68]. It is put forward that institutional perception of the difficulties to mental health innate in the graduate setting could be improved by this approach.

Cluster 4: The fourth category (aqua-colored) is broadly about human psychology in higher education systems in which some research is directed to the psychological experiences of graduate students. Studies in this cluster are about tools used in measuring Ph.D. students in research and appraising their value, for example, the “Perceived Stress” [9,12], the “MED NORD” [69,70], the “Satisfaction with Life”, the “Juniper Ph.D. student well-being” [67,71], and the “Motivation for a Ph.D.” (12, 67] scales. These tools have started to shed light concerning Ph.D. students’ mental health status. Further exploration through valid and dependable measures is necessary to identify ways to address mental health issues in this population.

Cluster 5: The fifth cluster (yellow-colored) is centered around the prevalence of mental stress activated by epidemics such as COVID-19 and their consequences on graduate students. The studies showed how epidemics could psychologically influence students’ health. For example, past reports showed that eruptions of transmittable diseases adversely impact students’ mental health, as seen in the past SARS outbreak [72,73]. The same effects have also been reported concerning the COVID-19 and mental health of students [74], which triggered more publication on this subject. The cluster also discusses interventions and implementation efforts on health promotion, positive psychology, and intrinsic motivation among graduate students. The studies here identified the importance of positive psychology and recommended it as an intervention strategy to avert mental health complications via extrinsic factors such as the educational atmosphere or the role of educators or mentors [75,76] or the intrinsic factors or qualities such as self-compassion and better self-regulation. It is believed that these may help guide the invention of mechanisms in higher education directed at advancing positive end results, such as student well-being and achievement.

### 4.9. Major Highlights of the Five Identified Clusters

The clusters looked at the well-being of Ph.D. students from several angles. Ph.D. students may leave the academic environment and research after finishing their studies due to emotional stress [77] or job insecurity if they cannot find a job they consider suitable after all the time spent to obtain the degree. Isolation and insecurity can harm Ph.D. students [78]. Graduate students’ educational chances and the apparent significance of their research are also influenced by academic communities [79]. Furthermore, conflicts originating from self-reflection and professional and familial roles impact this group of trainees [80,81]. In this perspective, the clusters recognized that Ph.D. students’ experiences are generally marked by stress, anxiety, and tension.

On the other hand, the clusters highlight potential positive variables in graduate students that could reduce the feelings of isolation and depression. The studies therein revealed that social support from friends and relatives is linked to less stress and higher satisfaction levels [82]. Likewise, feeling supported by the university is associated with a lower level of distress and higher levels of happiness [83].

In the presented materials, the cluster identified mental distress as the most common mental health concern among graduate students [84]. Furthermore, females reported more mental distress than males, with disparities rising as psychological distress increased. Gender disparities, on the other hand, are frequently caused by variables other than gender. Some writers hypothesized that the gender gap in mental disorders stems from a low probability of men’s mental difficulties being recognized and a lower possibility of studies with no difference between the genders being published.

A few institutional solutions, such as modifications in the evaluation and the system imbalance between effort and reward, are suggested by reducing or raising reward. Stress-management therapy, meditation. and yoga were useful in reducing mental distress among students [85]. Interventions at the organizational level, such as mentorship programs and extracurricular activities at the individual level, may help create a more supportive academic climate [86].

Further, researchers compared mental health in students before and during the COVID-19 outbreak and found increased emotional distress in China and India and a decline in mental health in the U.K. [87,88,89]. Creating effective interventions to promote mental health among graduate students requires further research on mental health triggers among graduate students, especially post-COVID-19.

### 4.10. Citation Analysis of Documents

The highest and most vastly cited papers offer information on the research trends and scientific progress in a particular field. The citation counts of mental health and well-being among graduate students’ publications were obtained from the *Scopus* database. Table 1 represents the list of the top 10 highly cited publications. These 10 papers cumulatively received 494 citations, which is 60.94% of the total citations. The top articles include “Work organization and mental health problems in Ph.D. students” and “The Ph.D. experience: A review of the factors influencing doctoral students’ completion, achievement, and well-being”, with 261 and 74 citations, published by the *Journal of Research Policy* and the *International Journal of Doctoral Policy*, respectively. Although the citation count is no scientific tool to measure publication, it is a valuable metric that identifies research parameters [90]. Table 1 might be beneficial to researchers in identifying the trends in mental health research, targeted journals, specialized organizations, and related authors.

**Figure 10:**
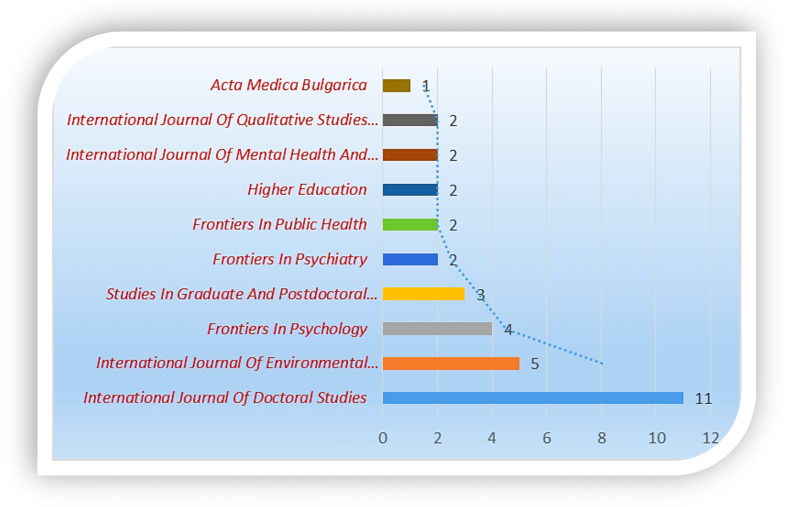
The top 10 journals with the highest number of publications (retrieved).

Further analysis of the top 10 journals with the highest published studies on the topic is presented in Figure 10. The *International Journal of Doctoral Studies,* the *International Journal of Environmental Research*, and *Frontiers in Psychology* account for the highest number of documents retrieved from the database.

## 5. Conclusions

The bibliometric study on the research on mental health and well-being among graduate students was conducted using the *Scopus* directory from 2012 to 2021. The literature trend indicates an increase in the awareness and urgency of the subject among researchers over time. This is reflected by the annual growing trends of publications in this research area. The interest in research in the mental health and well-being of graduate students has grown in the last decade and significantly in the last two years, indicating that this area is evolving and will continue progressing in the coming years. Secondly, the research over-represents European countries’ conceptual and disciplinary approaches and exhibits weak collaboration among diverse countries.

Study contributions

The study’s contributions have methodological, theoretical, and practical aspects. Methodologically, the use of bibliometrics brings a unique and currently explosive research technique to the body of research on graduate students’ mental health and well-being. The detailed explanation of the systemic process adopted enhances reliability and could guide new researchers on how to undertake research gap analysis using bibliometrics.

The study’s theoretical contributions lie in establishing the extant knowledge trend and foci. The study identified the keywords used in the collected data and organized them into clusters to generate a network visualization of recent research. By examining the four discovered clusters, readers can rapidly comprehend a history of published terms over time. These clusters are open to future research. This research has successfully mapped the focus over the last decade in four clusters and presented potential areas by analyzing the keyword clusters qualitatively. Therefore, the identified major themes in the clusters, the most frequented journals and critical terms, and the research gaps are vital for researchers in considering focus areas for graduate students’ mental health and well-being research.

Practically, this research provides evidence to hopefully instigate more discussions on policies and support systems for graduate students in their journey. Equal emphasis should be accorded to their quality of life and the number of graduates (completion rates) or research produced by doctoral and postdoctoral students.

Study limitations

This study has two limitations. First, given that there are other indexing databases available for bibliometrics, the results obtained from *Scopus* are not exhaustive. Articles indexed in other databases were excluded and therefore missed in the analysis. These could have added to the number of analyzed studies and insights. However, that *Scopus* is one of the largest databases for such analyses indicates that the findings are reasonably a reflection of reality. Secondly, the analyzed studies in this study are published through various models of open access because the authors observed that using these types allowed the collection of many articles for the relevant analyses. However, other articles not indexed in *Scopus* would also have added to the number of studies analyzed on the subject matter. In addition, given that some universities cover payment for open access, the exclusion of non-open-access articles may have influenced the differences reported between countries.

Future research

Further studies can include more databases and non-open-access publications to determine if different results may be obtained. Future work on this may explore the five research clusters shown in the network analysis (anxiety, outcome assessment, academic achievement, intervention study, distress syndrome) as different topics to contribute to the scholarly research needed and increase research output. Further studies are required to assess the frequency of mental health concerns; the function of key parameters, such as mentorship, gender, relationships, and support; and their intersection and their influence on mental health experiences among graduate students. More studies are required in communication and collaboration amongst researchers in this field as evidenced by the bibliometrics analysis’ weak collaboration results. Researchers can consider the significant differences between doctoral research programs in different countries (globally and across countries), and draw comparisons to promote innovative and relevant interventions.

Further, this review uncovered a dire need for further research that explores operational strategies capable of addressing the mental health predicament among graduate students through other innovative tools and methodologies. In addition, from the sampled literature, there is a gap in the lived experiences of graduate researchers, which could be the focus of further studies.

Further research is also needed to appraise and review higher educational institutions’ mental health policies and procedures. The analysis indicated that current mental health policies and procedures in research institutions are not yet well-understood. Therefore, reviewing current policies in place across institutions and cross-country would be valuable. In addition, it will be interesting to see if the trajectory of research in the past two years will hold post-COVID-19 and produce feasible policies to support graduate students in their journey with the adjustment to blended learning. Further research can explore these.

## Figures and Tables

**Table 1:** Top 10 publications with most citation on the subject of mental health and well-being among graduate students.

Publication Year	Document Title	Journal Title	<2017	2017	2018	2019	2020	2021	Total Citation
			7	11	30	91	142	213	494
2017	“Work organization and mental health problems in Ph.D. students”	*Research Policy*	0	4	24	54	77	102	261
2018	“The Ph.D. experience: A review of the factors influencing doctoral students’ completion, achievement, and well-being”	*International Journal of Doctoral Studies*	0	0	0	12	23	39	74
2018	“Doctoral students’ well-being: a literature review”	*International Journal of Qualitative Studies on Health and Well-being*	0	0	0	5	17	19	41
2013	“Experiences of disengagement—A study of doctoral students in the behavioral sciences”	*International Journal of Doctoral Studies*	7	7	4	8	3	2	31
2018	“A survey and a positive psychology intervention on French Ph.D. student well-being”	*International Journal of Doctoral Studies*	0	0	0	5	5	14	24
2018	“Effects of mental health interventions for students in higher education are sustainable over time: A systematic review and meta-analysis of randomized controlled trials”	*PeerJ*	0	0	2	2	9	10	23
2021	“Mental Health Status, Anxiety, and Depression Levels of Bangladeshi University Students During the COVID-19 Pandemic”	*International Journal of Mental Health and Addiction*	0	0	0	0	0	11	11
2019	“A comparative study of mental health and well-being among UK students on professional degree programmes”	*Journal of Further and Higher Education*	0	0	0	1	4	5	10
2017	“Psychological Well-being among Postgraduate Students”	*Acta Medica Bulgarica*	0	0	0	4	3	3	10
2020	“Promoting Mental Health and Psychological Thriving in University Students: A Randomized Controlled Trial of Three Well-Being Interventions”	*Frontiers in Psychiatry*	0	0	0	0	1	8	9

## Data Availability

Not applicable.
